# Lipoprotein-associated phospholipase A2 predicts cardiovascular death in patients on maintenance hemodialysis: a 7-year prospective cohort study

**DOI:** 10.1186/s12944-023-01991-0

**Published:** 2024-01-12

**Authors:** Lin Lin, Jie Teng, Yiqin Shi, Qiwen Xie, Bo Shen, Fangfang Xiang, Xuesen Cao, Xiaoqiang Ding, Xialian Xu, Zhen Zhang

**Affiliations:** 1 Department of Nephrology, Xiamen Branch, Zhongshan Hospital, Fudan University, Xiamen, Shanghai 200032 China; 2 Nephrology Clinical Quality Control Center of Xiamen, Xiamen, China; 3 Department of Nephrology, Zhongshan Hospital, Fudan University, No 180 Fenglin Road, Shanghai, 200032 China; 4 Shanghai Key Laboratory of Kidney and Blood Purification, Shanghai, China

**Keywords:** Hemodialysis, Lipoprotein-associated phospholipase A2, Dyslipidemia, Cardiovascular disease

## Abstract

**Background:**

Cardiovascular diseases (CVD) is the leading cause of death among maintenance hemodialysis patients, with dyslipidemia being a prevalent complication. The paradoxical relationship between cardiovascular outcomes and established lipid risk markers, such as low-density lipoprotein cholesterol (LDL-C), complicates lipid management in this population. This study investigated Lipoprotein-associated phospholipase A2 (Lp-PLA2), an emerging biomarker known for its proinflammatory and proatherogenic properties, as a potential cardiovascular prognostic marker in this cohort. In this context, the association between Lp-PLA2 levels and cardiovascular outcomes was evaluated, with the aim to facilitate more accurate stratification and identification of high-risk individuals.

**Methods:**

From August 2013 to January 2014, 361 hemodialysis patients were prospectively enrolled. Lp-PLA_2_ activity and laboratory measures at baseline were quantified. Comorbidities and medications were recorded. All patients were followed until the end of April, 2022. The individual and combined effects of Lp-PLA_2_ activity and LDL-C on patient outcomes were examined. The association between Lp-PLA_2_ activity and all-cause mortality, cardiovascular mortality, and major adverse cardiovascular events (MACEs) was analyzed.

**Results:**

The median Lp-PLA_2_ activity was 481.2 U/L. In subjects with Lp-PLA_2_ activity over 481.2 U/L, significantly higher total cholesterol (4.89 vs. 3.98 mmol/L; *P* < 0.001), LDL-C (3.06 vs. 2.22 mmol/L; *P* < 0.001), and apolipoprotein B (0.95 vs. 0.75 mmol/L; *P* < 0.001) were observed. Over a median follow-up of 78.1 months, 182 patients died, with 77 cases identified as cardiovascular death, 88 MACEs happened. Cardiovascular mortality and MACEs, but not all-cause mortality, were significantly increased in the high Lp-PLA2 group. Cox regression analyses showed that high Lp-PLA_2_ activity was associated with cardiovascular mortality and MACE occurrence. After comprehensive adjustment, high Lp-PLA_2_ activity was independently associated with cardiovascular mortality(as a dichotomous variable: HR:2.57, 95%CI:1.58,4.18, *P* < 0.001; as a continuous variable: HR:1.25, 95%CI:1.10,1.41, *P* = 0.001) and MACEs(as a dichotomous variable: HR:2.17, 95%CI:1.39,3.40, *P* = 0.001; as a continuous variable: HR:1.20, 95%CI:1.07,1.36, *P* = 0.002). When participants were grouped by median Lp-PLA2 activity and LDL-C values, those with high Lp-PLA_2_ and low LDL-C had the highest CV mortality. The addition of Lp-PLA2 significantly improved reclassification (as a dichotomous variable NRI = 42.51%, 95%CI: 5.0%,61.33%; as a continuous variable, NRI = 33.32%, 95% CI: 7.47%,56.21%).

**Conclusions:**

High Lp-PLA_2_ activity is an independent risk factor for cardiovascular mortality and MACEs occurrence in patients on hemodialysis. The combined measures of Lp-PLA_2_ and LDL-C help to identify individuals with a higher risk of cardiovascular death.

**Supplementary Information:**

The online version contains supplementary material available at 10.1186/s12944-023-01991-0.

## Introduction

Chronic Kidney Disease (CKD) stands as an independent risk factor for a diverse spectrum of cardiovascular diseases (CVDs), including coronary artery disease (CAD), heart failure, and stroke [1, 2]. The jeopardy amplifies notably among patients with End-Stage Kidney Disease (ESKD), who face a considerably heightened vulnerability to cardiovascular events and subsequent cardiovascular (CV)-associated mortality, despite the advancement of dialysis technology over recent decades. Notably, within the cohort of patients on maintenance hemodialysis (MHD), CV mortality constitutes 40%-50% of overall mortality, depicting an alarming elevation of 8.1-fold when compared with the general population [3–6].

Atherosclerosis, characterized by the formation of lipid-laden plaques within arterial walls, stands out as a pivotal contributor to the genesis of CVD [7]. While dyslipidemia is widely acknowledged as a risk factor for atherosclerosis and CVD in the general population [7, 8], patients with CKD/ESKD encounter an intricate nexus between atherosclerosis and determinants such as the retention of uremic toxins, fluid overload, inflammation, compromised endothelial function, and heightened oxidative stress [9].

Dyslipidemia, although highly prevalent, usually shows a distinct pattern in patients with CKD. The altered lipid profile in CKD includes elevated serum triglycerides, very low-density lipoprotein cholesterol (VLDL-C), reduced high-density lipoprotein cholesterol (HDL-C), and, less commonly, elevated concentrations of total cholesterol (TC) and low-density lipoprotein cholesterol (LDL-C) [10, 11]. Despite the abundant evidence supporting the utility of statin in high-risk populations, the available data inadequately substantiate the efficacy of conventional statin-centered lipid-lowering regimens within the dialysis population [12–14]. Therefore, the pursuit of emerging lipid biomarkers for CVD assumes critical importance in refining risk stratification among individuals undergoing dialysis. Such an endeavor holds significance not only for comprehending the mechanism of dyslipidemia but also for identifying potential therapeutic targets and improving strategies for lipid management in ESKD patients. One candidate biomarker is lipoprotein-associated phospholipase A2 (Lp-PLA_2_), an inflammatory serine lipase mainly produced by activated monocytes and macrophages that primarily binds to low-density lipoprotein (LDL) [15]. Lp-PLA_2_ is enriched in atherosclerotic plaques and has the biological activity of catalyzing the hydrolysis of phospholipids on the surface of LDLs, releasing lysophosphatidylcholine and oxidized fatty acids, triggering the inflammatory cascade, promoting and destabilizing the lipid core of atherosclerotic plaques, and inducing CV events [15–17]. In multiple clinical studies, elevated Lp-PLA_2_ levels have been found to be associated with an increased risk of CAD, stroke, and mortality [18–20]. Observational data suggested that Lp-PLA_2_ activity is predictive of cardiovascular events in dialyzed patients [21]. The reduction in Lp-PLA2 after atorvastatin treatment was associated with improved survival in MHD patients with type 2 diabetes [22]. However, it is important to underline that the exploration and the substantiation of the clinical utility of Lp-PLA2 among individuals with End-Stage Kidney Disease (ESKD) remain relatively limited.

Therefore, the purpose of the present study was to explore the prognostic value of Lp-PLA2 activity in patients on MHD.

## Methods

### Study population

From August 2013 to January 2014, patients on hemodialysis for over three months were recruited at the Hemodialysis Center, Zhongshan Hospital, Fudan University. All participants were followed up until the end of April 2022. Patients were excluded if they were under 18 years of age, had a history of failed kidney transplantation, had a history of myocardial infarction, or were diagnosed with active infection within three months before blood sampling. Demographic information, clinical information, and comorbidities were documented at baseline. The diagnosis of CAD was established by objective evidence, including noninvasive (e.g., stress electrocardiogram, echocardiography, coronary computed tomography angiography) and invasive tests (e.g., coronary angiography). Causes of death were identified and categorized according to the investigators' judgment. Follow-up events included all-cause mortality, CV mortality and major adverse cardiovascular events (MACEs). CV death included deaths from acute coronary syndrome, fatal arrhythmia or cardiac arrest, heart failure, stroke, or peripheral arterial disease (PAD). MACEs were defined as CV death, acute coronary syndrome, heart failure hospitalization, or stroke.

### Blood collection and biochemical measurements

Blood samples were collected before a regular mid-week hemodialysis session. Standard clinical laboratory methods were used to measure hemoglobin, serum albumin, prealbumin, creatinine, uric acid, homocysteine (Hcy), and lipid profiles. Lp-PLA_2_ activity was measured by a colorimetric test with a measuring range up to 2000 U/L and a limit of detection of 50.0 U/L (DiaSys Diagnostic Systems GmbH, Holzheim, Germany). The principle underlying this method involves the hydrolysis of the sn-position of 1-myristoyl-2-(4-nitrophenylsuccinyl)-sn-glycero-3-phosphocholine by Lp-PLA2, leading to the production of 4-nitrophenylsuccinate. Upon degradation in an aqueous medium, 4-nitrophenol forms, detectable via photometric analysis. The activity of Lp-PLA2 was determined based on changes in absorbance at specific wavelengths.

The reference range for the Lp-PLA2 assay was established through a study involving healthy individuals who adhered to specific clinical criteria. These criteria included: no history of CVDs or diabetes mellitus, not taking lipid-lowering drugs, not taking corticosteroids, triglyceride level below 1.70 mmol/L, TC level below 5.70 mmol/L, LDL-c level below 3.61 mmol/L, HDL-c above 0.91 mmol/L, fasting blood glucose level below 6.16 mmol/L and normal liver and kidney function. The serum reference intervals of Lp-PLA2 were determined as 230-728U/L for males, 194–640 U/L for females aged 18–49 years, and 208-698U/L for females aged 50–88 years [23].

### Statistical analysis

Continuous variables are expressed as the mean ± SD or median (interquartile range) as appropriate, and categorical variables are presented as numbers (percentage). Independent samples t tests were used to compare two groups of normally distributed data, whereas Mann‒Whitney U and chi-squared tests were performed for skewed and categorical data, respectively. Spearman's rank correlation testing was used to determine factors associated with Lp-PLA_2_ activity. Survival was estimated using the Kaplan‒Meier curve. Survival differences were examined using the log-rank test. The participants were grouped according to the median value of Lp-PLA2. The optimal cut-off values were determined by maximally selected rank statistics for Lp-PLA2 activity. Pairwise comparisons of survival curves were calculated, and *P* values were adjusted using the Benjamini‒Hochberg method. Univariable and multivariate Cox regression models were used to evaluate predictors of CV death and MACEs. Lp-PLA_2_ activity was included in Cox models as a continuous variable (Lp-PLA_2_ activity in units of U/L) or a dichotomous variable (stratified by the median value). The discriminative performance of Cox models was assessed by Harrel’s Concordance index or C-index. Net Reclassification Improvement (NRI) with the inclusion of Lp-PLA_2_ were calculated to appraise the incremental value of Lp-PLA_2_ activity. For NRI calculation, we employed the category-less approach, wherein only changes in predicted probability exceeding 5% were deemed relevant.

The restricted cubic splines with four knots (located at 0.05, 0.35, 0.65, 0.95) were used to explore the potential non-linear association of Lp-PLA_2_ activity with CV mortality and MACE occurrence.

Finally, we performed interaction analyses to assess the modifying effects of potential confounders on the relationship between Lp-PLA_2_ activity and investigated outcomes. For continuous variables, interaction effects were tested directly in their continuous scale to maximize the statistical efficiency. For categorical determinants, subgroup analyses were conducted to yield estimates specific to each subgroup. In addition, subgroup analyses were also conducted based on the median value of LDL-c, HDL-c and TG.

A* P* value of < 0.05 was considered statistically significant. Data analyses and visualization were performed with RStudio 2022.12.0 Build 353 and SPSS 26.0 (SPSS Inc., Chicago, IL, USA).

## Results

### Baseline characteristics of the study population

Participants' baseline characteristics are presented in Table 1. The study enrolled 361 patients on hemodialysis, of whom 223 (61.8%) were male. The median age was 61 (53,70) years old, and the dialysis vintage was 35.5 (17.8, 76.2) months. The prevalence of hypertension, diabetes, history of CAD and cerebral vascular accident (CVA) was 93.4%, 23.8%, 10.2%, and 11.4%, respectively. The median Lp-PLA_2_ activity observed within the study cohort was 481.2 U/L.

**Table 1:** Baseline demographic, clinical, and biochemical characteristics of the study population

	**All Patients (*n* = 361)**	**Low Lp-PLA**_**2 **_**(< 481.2U/L; *n* = 181)**	**High Lp-PLA**_**2**_** (> 481.2U/L; *n* = 180)**	***P***
Age(years)	61 (53,70)	62 (52,70)	60 (53,69)	0.474
Dialysis vintage(months)	35.5(17.8,76.2)	32.4(17.1,69.5)	37.5(20.1,79.7)	0.217
Gender, male (%)	223 (61.8)	121 (66.9)	102 (56.7)	**0.046**
Body mass index(kg/m^2^)	22.13 ± 3.50	22.02 ± 3.26	22.23 ± 3.74	0.578
Smoking history (%)	31 (8.6)	11 (6.1)	20(11.1)	0.088
Access (AVF), (%)	250 (69.3)	119 (65.7)	131(72.8)	0.148
Underlying kidney disease (%)				0.945
Chronic Glomerulonephritis	174(48.2)	87(48.1)	87(48.3)	
Diabetic nephropathy	41(11.4)	19(10.5)	22(12.2)	
Hypertensive nephropathy	49(13.6)	27(14.9)	22(12.2)	
Polycystic kidney disease	20(5.5)	11(6.1)	9(5.0)	
Others	14(3.9)	6(3.3)	8(4.4)	
Unknown	63(17.5)	31(17.1)	32(17.8)	
Comorbidity (%)				
Hypertension	337(93.4)	169(93.4)	168(93.3)	0.989
Diabetes	86(23.8)	45(24.9)	41(22.8)	0.642
Coronary artery disease	37(10.2)	24(13.3)	13(7.2)	0.059
Cerebrovascular event	41(11.4)	22(12.2)	19(10.6)	0.632
Medications (%)				
ACEI	73(20.2)	34(18.8)	39(21.7)	0.495
ARB	127(35.2)	59(32.6)	68(37.8)	0.303
CCB	268(74.2)	135(74.6)	133(73.9)	0.880
β-blockers	153(42.4)	78(43.1)	75(41.7)	0.784
Statin	66(18.3)	42(23.2)	24(13.3)	**0.015**
Aspirin	103(28.5)	48(26.5)	55(30.6)	0.396
Laboratory parameters				
Hemoglobin(g/L)	111.25 ± 15.8	110.6 ± 16.6	111.9 ± 14.9	0.459
Platelet(× 10^9^/L)	190.2 ± 69.5	192.8 ± 68.9	187.7 ± 70.1	0.490
White blood cell(× 10^9^/L)	6.46 ± 1.82	6.44 ± 1.73	6.48 ± 1.90	0.836
Neutrophil(× 10^9^/L)	4.31 ± 1.47	4.29 ± 1.38	4.33 ± 1.56	0.784
Lymphocyte(× 10^9^/L)	1.3(1.0,1.6)	1.3(1.0,1.6)	1.3(1.0,1.6)	0.752
Albumin(g/L)	38.63 ± 3.18	38.60 ± 3.28	38.66 ± 3.10	0.874
Pre-albumin(g/L)	0.28 ± 0.08	0.27 ± 0.09	0.29 ± 0.08	**0.005**
Urea(mmol/L)	24.6 ± 6.7	24.2 ± 6.6	25.1 ± 6.7	0.207
Creatinine(μmol/L)	1040.2 ± 301.2	1025.7 ± 296.1	1054.9 ± 306.4	0.357
Uric acid(mmol/L)	429.8 ± 79.5	429.8 ± 78.0	429.8 ± 81.2	0.995
Total cholesterol (mmol/L)	4.43 ± 1.12	3.98 ± 0.90	4.89 ± 1.14	** < 0.001**
LDL-c(mmol/L)	2.64 ± 0.99	2.23 ± 0.74	3.06 ± 1.05	** < 0.001**
HDL-c (mmol/L)	1.08 ± 0.33	1.09 ± 0.34	1.08 ± 0.33	0.700
Apo-B(g/L)	0.85 ± 0.24	0.75 ± 0.19	0.95 ± 0.24	** < 0.001**
Apo-A(g/L)	1.25 ± 0.27	1.25 ± 0.27	1.25 ± 0.27	0.856
Triglyceride(mmol/L)	1.55 ± 1.21	1.46 ± 1.10	1.64 ± 1.31	0.160
Homocysteine(μmol/L)	32.2(23.8,50.5)	29.8(22.5,51.9)	34.0(24.4,49.0)	0.374
Lp(a) (nmol/L)	202.0(105.0,366.5)	176.2(105.4,354.9)	208.5(103.2,398.0)	0.395
NT-proBNP(pg/ml)	3550(1857,7271)	3513(1789,7390)	3564(1896,7181)	0.860
Troponin T(ng/ml)	0.05(0.03,0.08)	0.046(0.032,0.079)	0.05(0.034,0.076)	0.498
hs-CRP (mg/L)	2.9(1.1,6.4)	2.64(1.03,6.78)	3.22(1.20,6.08)	0.590

*Abbreviations: ACEI* Angiotensin-converting-enzyme inhibitor, *Apo* Apolipoprotein, *ARB* Angiotensin II receptor blocker, *BMI* Body mass index, *CCB* Calcium channel blocker, *HDL-C* High-density lipoprotein cholesterol, *LDL-C* Low-density lipoprotein cholesterol, *Lp* Lipoprotein, *Hs-CRP* high-sensitivity C-reactive protein

The median serum Lp-PLA_2_ activity was used to classify the patients into two groups: the low Lp-PLA_2_ group and the high Lp-PLA_2_ group. Subjects in the high Lp-PLA_2_ group had significantly higher apo-B (0.95 vs. 0.75 g/L; *P* < 0.001), TC (4.89 vs. 3.98 mmol/L; *P* < 0.001), LDL-C (3.06 vs. 2.23 mmol/L; *P* < 0.001), and prealbumin (0.29 g/L vs. 0.27 g/L; *P* = 0.005). Additionally, a lower proportion of individuals within the high Lp-PLA_2_ group were observed to be recipients of statin therapy (13.3% vs. 23.2%; *P* = 0.015). However, no statistically significant disparities were identified between the high Lp-PLA_2_ group and the group with lower Lp-PLA_2_ activity concerning all other demographic, clinical, and biochemical parameters. The correlation analyses findings are detailed in Table 2.

**Table 2:** Correlation analyses of variables associated with Lp-PLA_2_ activity

	r	*P* value
Age(yr)	-0.058	0.273
Male	0.020	0.700
Body mass index(kg/m^2^)	0.088	0.097
Smoking history	0.067	0.204
Hypertension	0.017	0.747
Diabetes	-0.013	0.810
Statin	-.146	0.005
Hemoglobin(g/L)	0.032	0.549
Albumin(g/L)	-0.002	0.966
Urea (mmol/L)	0.007	0.898
Serum creatinine(μmol/L)	0.087	0.099
Uric acid (mmol/L)	-0.012	0.827
Total cholesterol (mmol/L)	.463	< 0.001
LDL-c (mmol/L)	.443	< 0.001
HDL-c (mmol/L)	-0.033	0.526
Apo-B (g/L)	.445	< 0.001
Apo-A (g/L)	-0.030	0.571
Triglyceride (mmol/L)	.149	0.004
Lp(a) (nmol/L)	0.021	0.693
hs-CRP (mg/L)	0.025	0.642

*Apo* Apolipoprotein, *HDL-C* High-density lipoprotein cholesterol, *LDL-C* Low-density lipoprotein cholesterol, *Lp* Lipoprotein, *hs-CRP* High-sensitivity C-reactive protein

### Lipid profile and patient outcomes

All enrolled patients were followed until April 1st, 2022. The median follow-up was 78.1 (34.7, 104.5) months. During follow-up, 182 patients died, 14 patients received kidney transplantation, 1 patient was converted to peritoneal dialysis and 30 patients were transferred to other centers.

Mortality incidents were primarily attributed to CVDs (*n* = 77; 42.3%), followed by infection (*n* = 42; 23.1%), cancer (*n* = 17; 9.3%), gastrointestinal bleeding (*n* = 10; 5.5%), and other causes (*n* = 36; 19.8%). CV death comprised 18 cases of acute coronary syndrome, 16 cases of hemorrhagic stroke, 15 cases of heart failure, 13 cases of cardiac arrests/fatal arrhythmia, 13 cases of ischemic stroke, and 2 cases of PADs.

Eighty-eight patients within the cohort experienced major adverse cardiovascular events (MACEs). This set of MACEs encompassed 21 CV deaths, 16 cases of acute coronary syndrome, 9 cases of heart failure hospitalization, 21 cases of ischemic stroke, and 21 cases of hemorrhagic stroke.

**Fig. 1:**
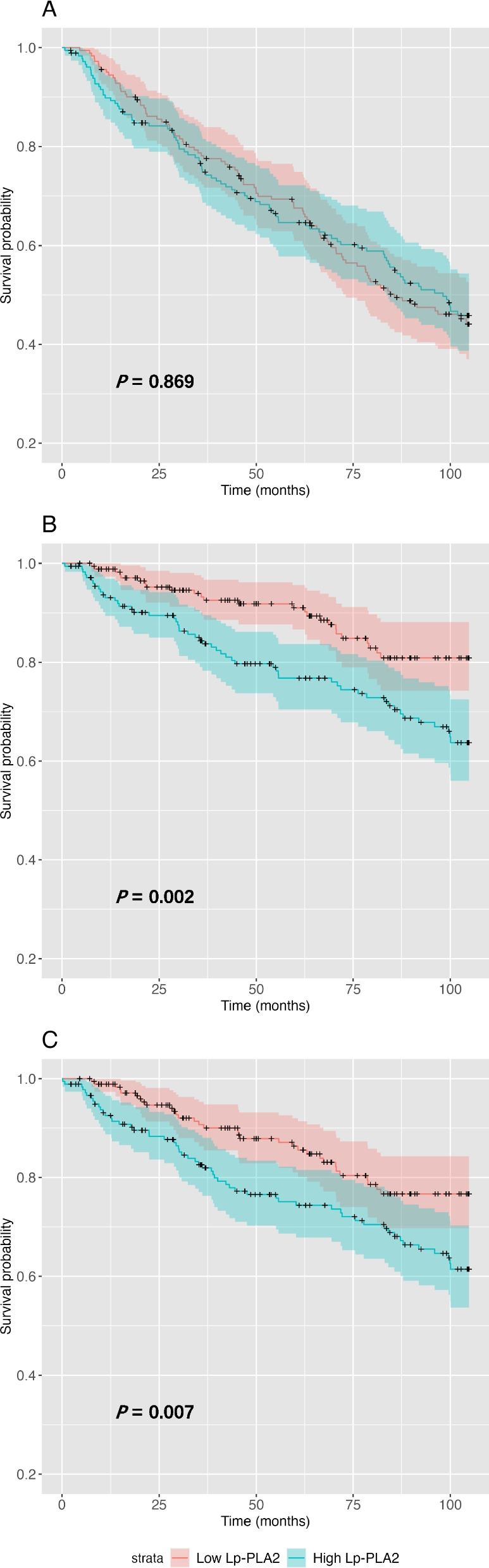
Kaplan‒Meier curves of (**A**) all-cause mortality, (**B**) CV mortality and (**C**) MACEs stratified by low and high Lp-PLA_2_ groups

**Fig. 2:**
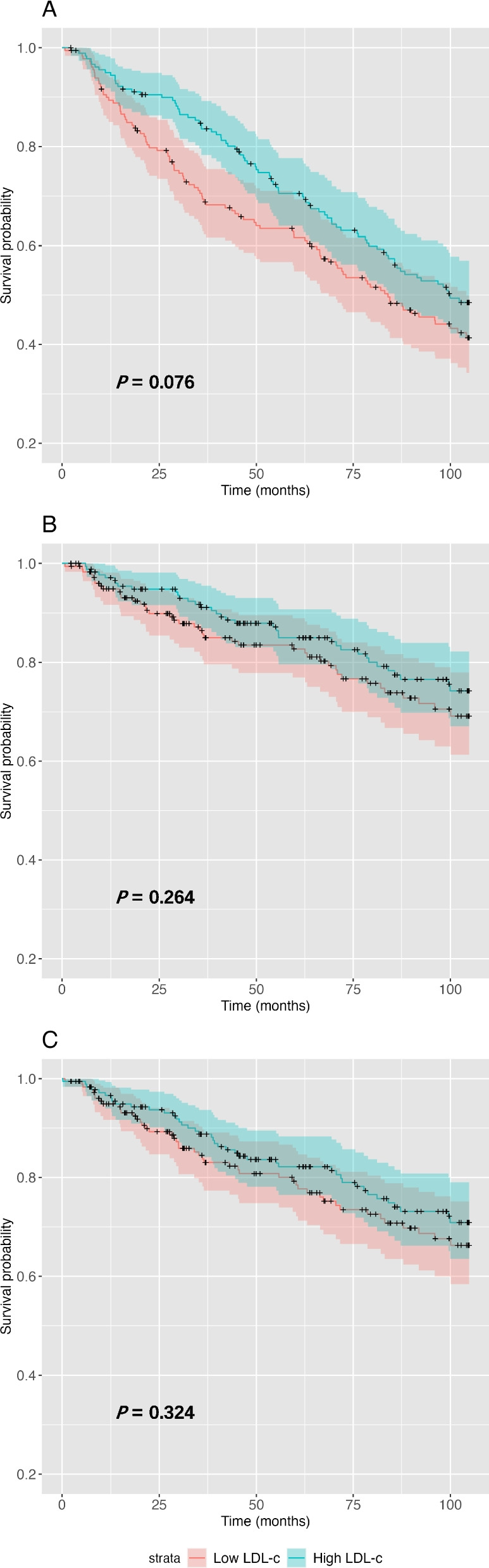
Kaplan‒Meier curves of (**A**) all-cause mortality, (**B**) CV mortality and (**C**) MACEs stratified by low and high LDL-C groups

When analyzed with the Kaplan‒Meier method, CV deaths and MACEs were significantly increased in the high Lp-PLA_2_ group compared with the low Lp-PLA_2_ group (CV death: *P* = 0.002; MACEs: *P* = 0.007). Whereas all-cause mortality was comparable between the two groups (*P* = 0.869), as depicted in Fig. 1. As illustrated in Fig. 2, when patients were stratified according to their LDL levels, no significant differences were observed in terms of all-cause mortality (*P* = 0.076), CV mortality (*P* = 0.264), or occurrence of MACEs (*P* = 0.324).

**Fig. 3:**
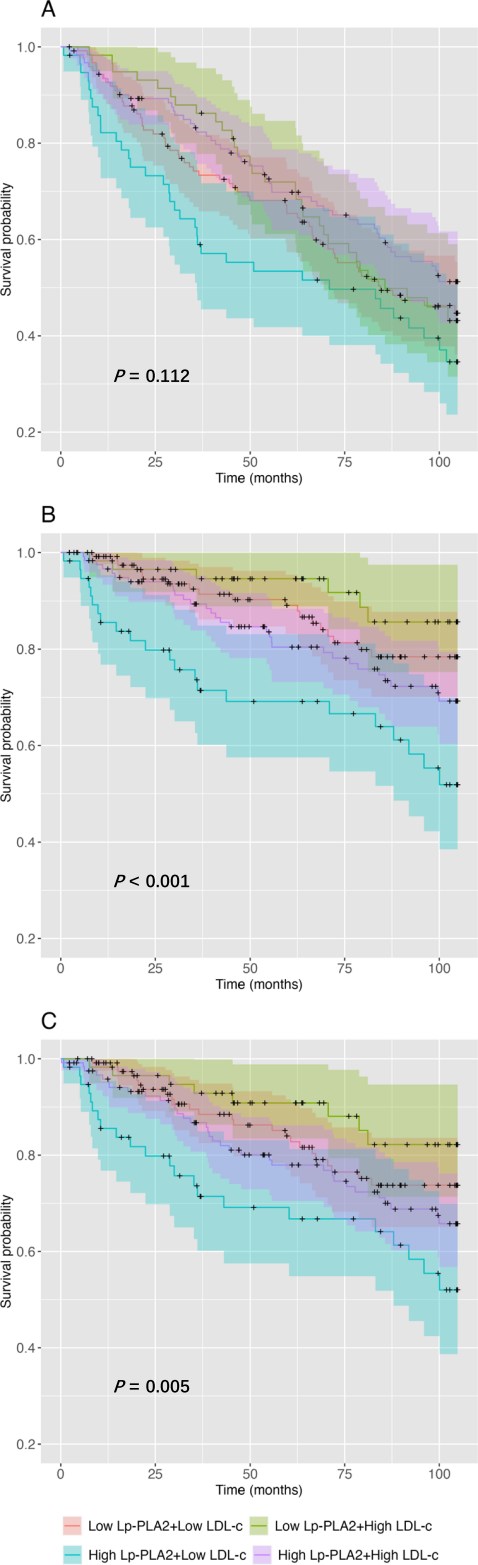
Kaplan‒Meier curves of (**A**) all-cause mortality, (**B**) CV mortality and (**C**) MACEs stratified by medians of both Lp-PLA_2_ and LDL-C

As illustrated in Fig. 3, subjects were classified into four groups based on the median values of both Lp-PLA_2_ and LDL-C. All-cause mortality was not significantly different across the four groups (*P* = 0.112). CV death and MACE occurrence were significantly different among the four groups (CV mortality, *P* < 0.001; MACE occurrence, *P* = 0.005). Specifically, the highest incidence of CV mortality was observed within the subset characterized by high Lp-PLA2 activity with low LDL-C levels (vs. Low Lp-PLA_2_ + Low LDL-C, *P* = 0.002; vs. Low Lp-PLA_2_ + High LDL-C, *P* = 0.002; vs. High Lp-PLA2 + High LDL-C, *P* = 0.04).

With respect to the occurrence of MACEs, the subgroup featuring high Lp-PLA2 activity and low LDL-C levels demonstrated a significantly elevated incidence in comparison to both the subgroup with low Lp-PLA2 activity and low LDL-C levels (*P* = 0.021), as well as the subgroup with low Lp-PLA2 activity and high LDL-C levels (*P* = 0.010). In contrast, pairwise comparisons showed no significant between-group differences in terms of all-cause mortality.

**Fig. 4:**
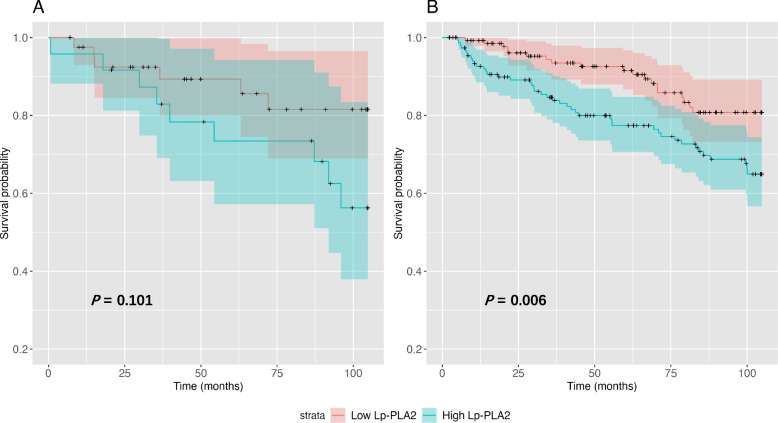
Kaplan‒Meier curves of CV mortality stratified by low and high Lp-PLA_2_ groups in the setting of receiving statin therapy (**A**) or not (**B**)

**Fig. 5:**
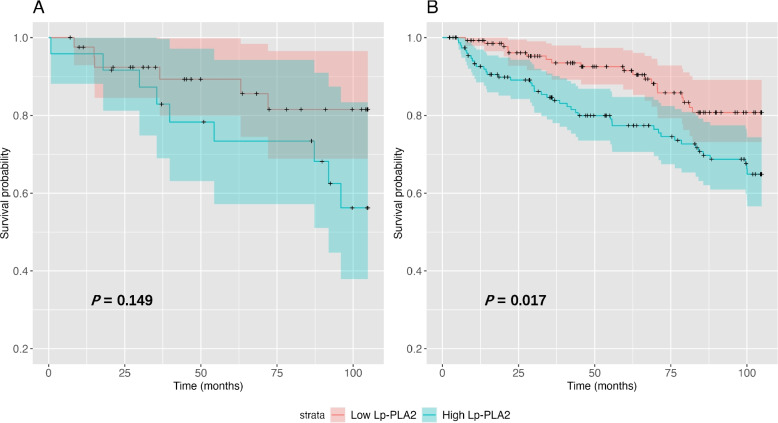
Kaplan‒Meier curves of MACEs stratified by low and high Lp-PLA_2_ groups in the setting of receiving statin therapy (**A**) or not (**B**)

The analysis further extends by stratifying subjects according to statin use. Among those not receiving statins, high Lp-PLA_2_ activity was associated with higher CV mortality (*P* = 0.006) and an increased MACE occurrence (*P* = 0.017). Intriguingly, this correlation was observed to be attenuated among those individuals under statin therapy (CV death: *P* = 0.101; MACE: *P* = 0.149), as illustrated in Figs. 4 and 5. When Lp-PLA_2_ and LDL-c were used in combination, the all-cause mortality, CV mortality and MACE occurrence were comparable across four strata among statin users. Pairwise comparisons showed no significant between-group differences in terms of all-cause mortality, CV mortality and MACE occurrences. In non-statin users, CV mortality and MACE occurrence were significantly different among the four strata (CV mortality, *P* = 0.002; MACE occurrence, *P* = 0.018), while all-cause mortality was comparable (*P* = 0.154). (Figures S1 & S2).

Optimal Lp-PLA_2_ cut-off values for predicting outcomes were determined by maximally selected rank statistics. The cut-off for all-cause mortality, CV mortality and MACE were 358RU/mL, 444RU/mL and 450 RU/mL respectively.

Univariate Cox proportional hazard analyses were conducted to assess the individual associations between various variables and the outcomes, and hazard ratios (HRs) were calculated. As shown in Table 3, age(HR = 1.05, *P* < 0.001), AVF as vascular access(HR 0.48, *P* = 0.002), Lp-PLA_2_ > 481.2 U/L (HR 2.15, *P* = 0.002), Lp-PLA_2_ activity (HR 1.18,* P* = 0.004), N-terminal pro b-type natriuretic peptide (NT-proBNP) (HR 2.08, *P* = 0.001), high sensitivity cardiac troponin T (hs-cTnT) (HR 7.30, *P* < 0.001), albumin (HR 0.94,* P* = 0.043), Serum creatinine (HR 0.99, *P* < 0.001), Uric acid (HR 0.99, *P* = 0.009), history of diabetes (HR 2.23, *P* = 0.001), history of CAD (HR 2.81, *P* < 0.001) were significantly associated with CV death.

**Table 3:** Univariate Cox proportional hazard model of variables associated with CV death and MACE in the study population

Variable	CV death		MACE	
	HR(95% CI)	*P* value	HR(95% CI)	*P* value
Age(years)	1.05(1.03–1.07)	** < 0.001**	1.06(1.04–1.08)	** < 0.001**
Dialysis vintage (month)	0.99(0.98–1.00)	0.06	0.99(0.99–1.00)	0.096
Sex (male vs female)	1.34(0.86–2.11)	0.2	1.22(0.80–1.87)	0.349
Vascular access (catheter vs arteriovenous fistula)	0.48(0.30–0.76)	**0.002**	0.41(0.26–0.62)	** < 0.001**
Body mass index (kg/m2)	1.07(1.00–1.13)	**0.049**	1.05(0.99–1.11)	0.111
Smoking history (%)	1.38(0.66–2.87)	0.387	1.19(0.58–2.47)	0.634
Hypertension	1.55(0.21–11.47)	0.67	1.98(0.63–6.27)	0.244
Diabetes	2.23(1.39–3.57)	**0.001**	2.02(1.29–3.16)	**0.002**
Coronary artery disease	2.81(1.59–4.94)	** < 0.001**	2.57(1.50–4.43)	**0.001**
Cerebrovascular event	1.24(0.59–2.58)	0.568	1.21(0.61–2.42)	0.58
ACEI	0.65(0.36–1.17)	0.152	0.61(0.34–1.07)	0.085
ARB	0.82(0.51–1.32)	0.417	0.75(0.48–1.19)	0.222
CCB	0.84(0.51–1.38)	0.484	0.93(0.58–1.51)	0.783
β-blockers	0.86(0.55–1.36)	0.521	0.85(0.55–1.30)	0.444
Statin	1.07(0.61–1.87)	0.828	1.13(0.68–1.90)	0.637
Aspirin	1.12(0.69–1.82)	0.656	1.04(0.66–1.65)	0.867
Lp-PLA_2_ > 481.2U/L	2.15(1.33–3.46)	**0.002**	1.81(1.17–2.80)	**0.007**
Lp-PLA_2 _(per 100 U/L)	1.18(1.05–1.32)	**0.004**	1.14(1.02–1.27)	**0.018**
Hemoglobin (g/L)	1.00(0.99–1.01)	0.984	0.99(0.99–1.01)	0.929
Platelet (× 10^9^/L)	1.00(0.99–1.01)	0.408	1.00(0.99–1.00)	0.402
Albumin (g/L)	0.94(0.88–0.99)	**0.043**	0.94(0.88–0.99)	**0.046**
Pre-albumin (per 0.1 g/L)	0.11(0.01–1.81)	0.122	0.08(0.01–1.11)	0.06
Urea (mmol/L)	0.98(0.96–1.02)	0.506	0.99(0.95–1.01)	0.259
Serum creatinine (μmol/L)	0.99(0.98–0.99)	** < 0.001**	0.99(0.98–1.00)	**0.001**
Uric acid (mmol/L)	0.99(0.98–0.99)	**0.009**	0.99(0.98–0.99)	**0.01**
Total cholesterol (mmol/L)	1.015(0.83–1.24)	0.878	1.02(0.86–1.21)	0.827
LDL-c (mmol/L)	0.99(0.79–1.14)	0.931	0.98(0.80–1.21)	0.881
HDL-c (mmol/L)	1.17(0.61–2.23)	0.646	1.04(0.56–1.93)	0.901
Apo-B(g/L)	0.91(0.35–2.34)	0.847	1.02(0.42–2.44)	0.971
Apo-A(g/L)	0.76(0.32–1.79)	0.529	0.75(0.34–1.67)	0.483
Triglyceride (mmol/L)	0.98(0.81–1.19)	0.848	1.02(0.86–1.21)	0.827
Homocysteine (μmol/L)	0.53(0.21–1.30)	0.166	0.74(0.33–1.66)	0.467
Lipoprotein(a) (nmol/L)	1.21(0.73–2.00)	0.465	1.40(0.86–2.25)	0.172
NT-proBNP (pg/ml)	2.08(1.33–3.27)	**0.001**	1.87(1.22–2.85)	**0.004**
Cardiac troponin T (ng/ml)	7.30(3.27–16.32)	** < 0.001**	6.52(3.06–13.87)	** < 0.001**
High-sensitivity C-reactive protein(mg/L)	1.44(0.97–2.15)	0.072	1.43(0.98–2.07)	0.063

*ACEI* Angiotensin-converting-enzyme inhibitor, *ARB* Angiotensin II receptor blocker, *CCB* Calcium channel blocker, *HDL-c* High-density lipoprotein cholesterol, *LDL-c* Low-density lipoprotein cholesterol

Age (HR 1.06, *P* < 0.001), AVF as vascular access(HR = 0.41, *P* < 0.001), Lp-PLA_2_ > 481.2 U/L (HR 1.81, *P* = 0.007), Lp-PLA_2_ (HR = 1.41, *P* = 0.018), NT-proBNP (HR 1.87, *P* = 0.004), hs-cTnT (HR 6.52, *P* < 0.001), albumin (HR = 0.94, *P* = 0.046), Serum creatinine (HR 0.99, *P* = 0.001), Uric acid (HR 0.99, *P* = 0.01), history of diabetes (HR 2.02, *P* = 0.002), history of CAD (HR 2.57, *P* = 0.001) were significantly associated with MACEs (Table 3).

**Fig.6:**
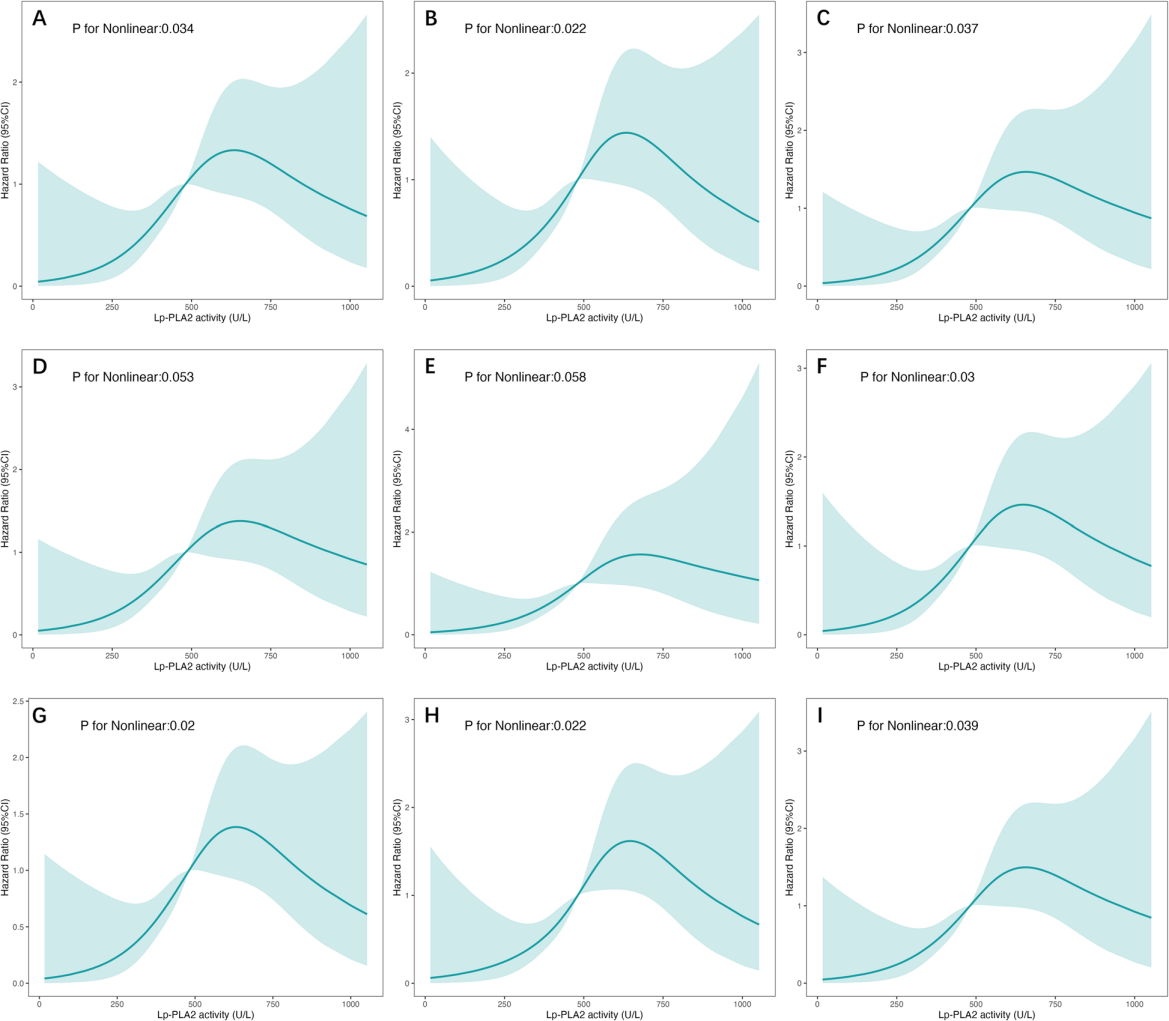
Association of Lp-PLA_2_ activity and cardiovascular mortality**.** Restricted cubic spline analyses to assess nonlinear association between Lp-PLA_2_ activity and cardiovascular mortality. Covariates for adjustment **A**. unadjusted **B**. age, dialysis vintage, sex, BMI, vascular access **C**. history of hypertension, diabetes, coronary artery disease and cerebrovascular event **D**. hemoglobin, creatine, uric acid, albumin **E**. HDL-c, LDL-c, triglyceride, apoB, apoA **F**. hs-cTnT, NT-proBNP, hs-CRP **G**. use of ACEI, ARB, statin, asprin, CCB, beta blocker **H**. age, dialysis vintage, vascular access, BMI, history of diabetes, coronary artery disease, albumin, hs-CRP, creatine, uric acid, hs-cTNT, NT-proBNP I. age, BMI, hs-cTnT, creatine, NT-proBNP

**Fig.7:**
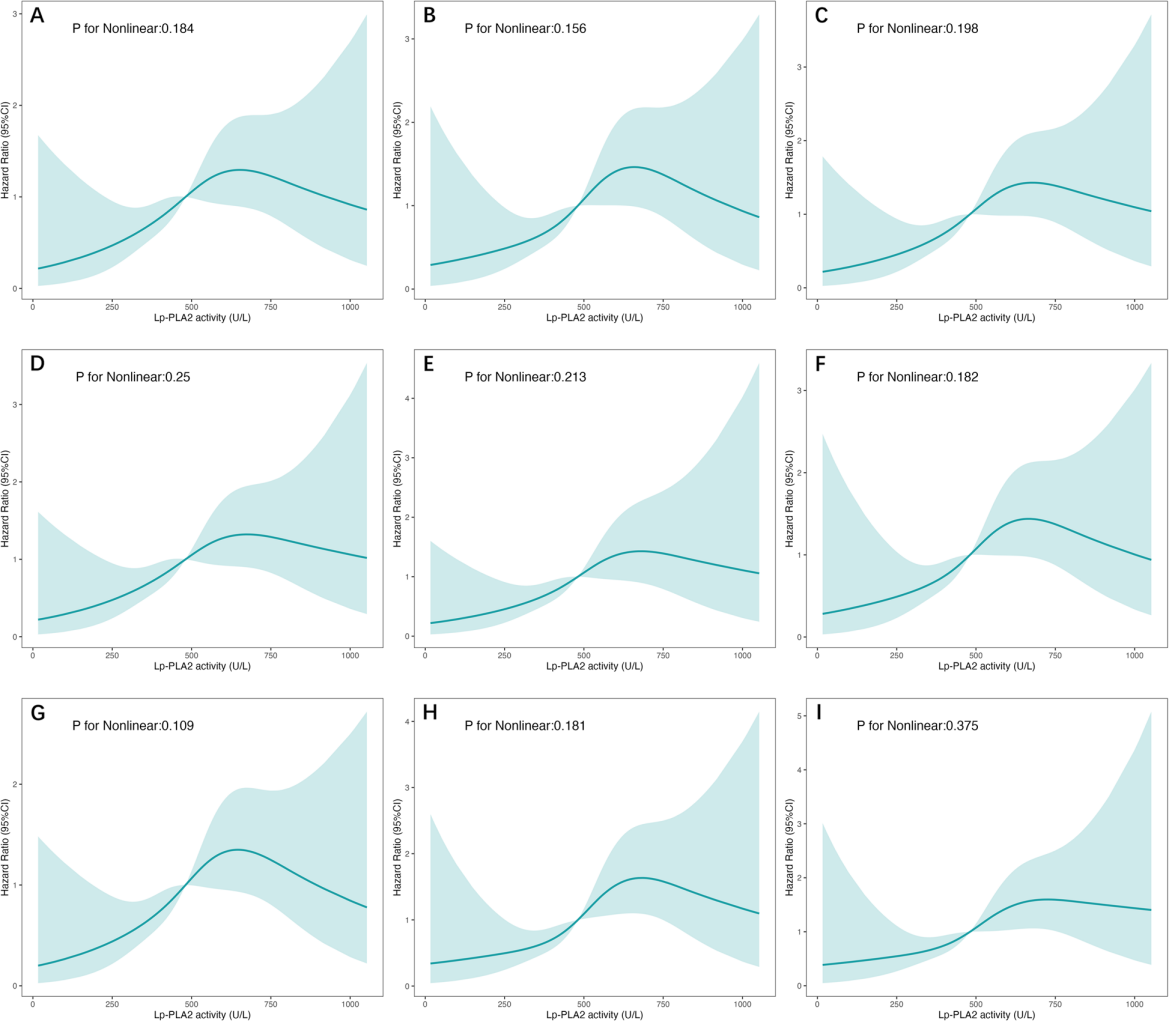
Association of Lp-PLA_2_ activity and MACEs**.** Restricted cubic spline analyses to assess nonlinear association between Lp-PLA_2_ activity and MACEs. Covariates for adjustment **A**. unadjusted **B**. age, dialysis vintage, sex, BMI, vascular access **C**. history of hypertension, diabetes, coronary artery disease and cerebrovascular event **D**. hemoglobin, creatine, uric acid, albumin **E**. HDL-c, LDL-c, triglyceride, apoB, apoA **F**. hs-cTnT, NT-proBNP, hs-CRP **G**. use of ACEI, ARB, statin, asprin, CCB, beta blocker **H**. age, dialysis vintage, vascular access, BMI, history of diabetes, coronary artery disease, albumin, hs-CRP, creatine, uric acid, hs-cTNT, NT-proBNP **I**. age, vascular access, history of diabetes, hs-cTnT

Seven multivariate Cox models were applied to examine the association of Lp-PLA_2_ with outcomes, each adjusting for baseline demographic data, primary comorbidities, key laboratory measures, lipid profiles, cardiac and inflammatory biomarkers, as well as medications. The final model included all the confounders with a p-value below 0.10 in the univariate Cox model, using both the 'enter' method and the 'stepwise' approach for adjustments. In all the analyzed models, whether Lp-PLA_2_ was treated as a categorical variable (stratified by median values) or as a continuous variable (measuring HR for every 100U/L increment), Lp-PLA_2_ consistently emerged as a significant risk factor for CV death and MACE occurrence. The results are detailed in Table 4. In summary, after comprehensive adjustment, Lp-PLA_2_ was significantly associated with CV death when assessed as a continuous variable (HR 1.25, 95%CI 1.10–1.41, *P* = 0.001) or dichotomous variable (HR 2.57, 95%CI 1.58–4.18, *P* < 0.001). Lp-PLA_2_ was also significantly associated with MACE either as a continuous variable (HR 1.20, 95%CI 1.07–1.36, *P* = 0.002) or dichotomous variable (HR 2.17 95%CI 1.39–3.40, *P* = 0.001). Restricted Cubic Splines (RCS) analyses suggested a non-linear relationship between Lp-PLA_2_ and both CV mortality and MACE occurrence. However, this association did not achieve statistical significance in all models. Upon visual examination, there was a discernible trend where HR consistently rose in tandem with increasing Lp-PLA_2_ until it reached a threshold of approximately 600–700 U/L. Beyond this threshold, the HR exhibited a trend for decline. This trend is graphically represented in the Figs. 6 and 7.

**Table 4:** Multivariate Cox models of CV death and MACE in MHD patients

Model	Cardiovascular death		MACE	
	**HR (95% CI)**	***P***	**HR (95% CI)**	***P***
**Lp-PLA**_**2**_** (as a dichotomous variable)**				
Unadjusted	2.12(1.32–3.42)	0.002	1.81(1.17–2.80)	0.007
Model 1	2.19(1.35–3.54)	0.001	2.00(1.29–3.10)	0.002
Model 2	2.36(1.46–3.84)	0.001	2.14(1.37–3.34)	0.001
Model 3	2.38(1.46–3.90)	0.001	2.11(1.34–3.30)	0.001
Model 4	3.03(1.74–5.28)	< 0.001	2.34(1.42–3.87)	0.001
Model 5	3.11(1.79–5.41)	< 0.001	2.37(1.44–3.92)	0.001
Model 6	3.16(1.82–5.52)	< 0.001	2.44(1.47–4.05)	0.001
Model 7a	2.57(1.58–4.18)	< 0.001	2.17(1.39–3.40)	0.001
Model 7b	2.48(1.53–4.03)	< 0.001	2.12(1.36–3.31)	0.001
**Lp-PLA**_**2**_**(per 100U/L) (as a continuous variable)**				
Unadjusted	1.18(1.05–1.32)	0.004	1.01(1.00–1.02)	0.018
Model 1	1.19(1.06–1.34)	0.003	1.17(1.04–1.31)	0.008
Model 2	1.22(1.08–1.38)	0.001	1.19(1.06–1.34)	0.004
Model 3	1.23(1.08–1.39)	0.002	1.19(1.05–1.38)	0.005
Model 4	1.42(1.18–1.70)	< 0.001	1.26(1.07–1.48)	0.002
Model 5	1.45(1.20–1.74)	< 0.001	1.28(1.08–1.50)	0.003
Model 6	1.47(1.21–1.78)	< 0.001	1.29(1.09–1.53)	0.003
Model 7a	1.25(1.10–1.41)	0.001	1.20(1.07–1.36)	0.002
Model 7b	1.24(1.10–1.40)	0.001	1.20(1.07–1.35)	0.002

Covariates adjusted for CV death: age, dialysis vintage, vascular access, BMI, history of diabetes, coronary artery disease, albumin, hs-CRP, creatine, uric acid, hs-cTNT, NT-proBNP. Covariates adjusted for MACE: age, dialysis vintage, vascular access, history of diabetes, coronary artery disease, history of taking ACEI, albumin, prealbumin, hs-CRP, creatine, uric acid, hs-cTNT, NT-proBNP

Adjusted variables for each model

a: using “enter” approach

b: using “backward stepwise” approach

Model 1: age, dialysis vintage, sex, BMI, vascular access

Model 2: history of hypertension, diabetes, coronary artery disease and cerebrovascular event

Model 3: hemoglobin, creatine, uric acid, albumin

Model 4: HDL-c, LDL-c, triglyceride, apoB, apoA

Model 5: hs-cTnT, NT-proBNP, hs-CRP

Model 6: use of ACEI, ARB, statin, asprin, CCB, beta blocker

Model 7: hierarchically selected covariates (*P* < 0.1 in the univariate Cox model)

Results of subgroup and interaction analyses were detailed in Table 5 and Table 6. Notably, interaction tests across all covariates were consistently non-significant (*P* > 0.05), suggesting no evidence of modifying effects of the covariates on the association between Lp-PLA_2_ activity and both CV death and MACE occurrence.

**Table 5:** Interaction analyses of Lp-PLA2 activity with CV death and MACE occurrence across different subgroups

CV death	Events/Total	Unadjusted		*P* for Interaction	Adjusted		*P* for Interaction
		**HR (95% CI)**	***P***		**HR (95% CI)**	***P***	
*Lp-PLA*_*2*_* (as a dichotomous variable)*							
Sex				0.247			0.385
Male	43/223	1.68(0.91–3.11)	0.096		2.06(1.04–4.08)	0.038	
Female	34/138	3.01(1.36–6.67)	0.007		3.95(1.68–9.23)	0.002	
Age				0.329			0.53
≥ 60	57/198	2.24(1.29–3.89)	0.004		2.65(1.49–4.69)	0.001	
< 60	20/163	2.21(0.85–5.75)	0.105		2.70(0.91–7.99)	0.074	
History of diabetes				0.313			0.205
Yes	27/86	3.06(1.34–7.02)	0.008		3.99(1.67–9.54)	0.002	
No	50/275	1.87(1.04–3.35)	0.037		2.21(1.20–4.06)	0.011	
History of CAD				0.399			0.774
Yes	15/37	3.27(1.16–9.23)	0.025		2.05(0.65–6.50)	0.221	
No	62/324	2.26(1.31–3.92)	0.004		2.70(1.54–4.73)	0.001	
LDL-c (mmol/L)				0.821			0.733
≥ 2.45	36/181	2.44(1.01–5.86)	0.046		2.68(1.06–6.78)	0.037	
< 2.45	41/180	2.74(1.48–5.07)	0.001		3.81(1.88–7.71)	< 0.001	
HDL-c (mmol/L)				0.771			0.470
≥ 1.03	39/184	1.98(1.03–3.81)	0.04		2.04(1.02–4.09)	0.045	
< 1.03	38/177	2.30(1.14–4.63)	0.02		3.50(1.65–7.44)	0.001	
TG (mmol/L)				0.745			0.576
≥ 1.29	39/182	2.39(1.14–5.05)	0.022		3.30(1.48–7.37)	0.004	
< 1.29	38/179	2.03(1.06–3.86)	0.032		2.43(1.25–4.74)	0.009	
*Lp-PLA*_*2*_* (as a continuous variable)(per 100U/L)*							
Sex				0.169			0.25
Male	43/223	1.09(0.93–1.28)	0.271		1.20(0.99–1.46)	0.064	
Female	34/138	1.29(1.09–1.52)	0.003		1.33(1.12–1.59)	0.002	
Age				0.854			0.712
≥ 60	57/198	1.19(1.04–1.35)	0.1		1.22(1.06–1.40)	0.006	
< 60	20/163	1.21(0.97–1.53)	0.096		1.28(0.99–1.65)	0.053	
History of diabetes				0.11			0.086
Yes	27/86	1.47(1.13–1.90)	0.004		1.53(1.17–2.01)	0.002	
No	50/275	1.16(1.01–1.33)	0.041		1.21(1.04–1.41)	0.015	
History of CAD				0.379			0.688
Yes	15/37	1.31(0.99–1.74)	0.059		1.09(0.73–1.64)	0.667	
No	62/324	1.19(1.06–1.35)	0.007		1.27(1.11–1.46)	0.001	
LDL-c (mmol/L)				0.317			0.061
≥ 2.45	36/181	1.15(0.96–1.38)	0.116		1.16(0.95–1.42)	0.135	
< 2.45	41/180	1.30(1.12–1.52)	0.001		1.62(1.29–2.04)	< 0.001	
HDL-c (mmol/L)				0.923			0.122
≥ 1.03	39/184	1.17(1.00–1.37)	0.047		1.21(1.01–1.46)	0.043	
< 1.03	38/177	1.20(1.01–1.41)	0.041		1.33(1.11–1.60)	0.002	
TG (mmol/L)				0.574			0.879
≥ 1.29	39/182	1.17(1.00–1.36)	0.048		1.25(1.05–1.49)	0.011	
< 1.29	38/179	1.25(1.03–1.52)	0.026		1.35(1.08–1.68)	0.008	
**MACEs**	**Events/Total**	**Unadjusted**		***P* for Interaction**	**Adjusted**		***P* for Interaction**
		**HR (95% CI)**	***P***		**HR (95% CI)**	***P***	
*Lp-PLA*_*2*_* (as a dichotomous variable)*							
Sex				0.105			0.311
Male	51/223	1.35(0.78–2.35)	0.285		1.72(0.93–3.18)	0.087	
Female	37/138	2.91(1.37–6.17)	0.005		3.99(1.78–8.94)	0.001	
Age				0.595			0.246
≥ 60	67/198	1.80(1.10–2.95)	0.019		2.10(1.26–3.49)	0.004	
< 60	21/163	2.39(0.93–6.15)	0.072		3.10(1.02–9.41)	0.047	
History of diabetes				0.352			0.282
Yes	29/86	2.50(1.16–5.40)	0.019		2.77(1.25–6.13)	0.012	
No	59/275	1.64(0.97–2.77)	0.068		1.97(1.14–3.41)	0.016	
History of CAD				0.332			0.914
Yes	16/37	2.81(1.03–7.62)	0.043		1.86(0.54–6.43)	0.325	
No	72/324	1.88(1.15–3.07)	0.012		2.25(1.35–3.75)	0.002	
LDL-c (mmol/L)				0.965			0.993
≥ 2.45	42/181	2.14(0.99–4.61)	0.054		2.39(1.05–5.44)	0.039	
< 2.45	46/180	2.18(1.22–3.88)	0.008		2.59(1.36–4.95)	0.004	
HDL-c (mmol/L)				0.792			0.540
≥ 1.03	43/184	1.71(0.93–3.15)	0.087		1.72(0.90–3.29)	0.102	
< 1.03	45/177	1.93(1.04–3.59)	0.038		2.77(1.41–5.46)	0.033	
TG (mmol/L)				0.956			0.739
≥ 1.29	46/182	1.85(0.98–3.52)	0.059		2.38(1.19–4.76)	0.014	
< 1.29	42/179	1.80(0.98–3.30)	0.059		1.95(1.02–3.75)	0.044	
*Lp-PLA*_*2*_* (as a continuous variable)(per 100U/L)*							
Sex				0.055			0.409
Male	51/223	1.03(0.89–1.20)	0.692		1.12(0.93–1.34)	0.24	
Female	37/138	1.28(1.09–1.51)	0.002		1.35(1.13–1.61)	< 0.001	
Age				0.343			0.804
≥ 60	67/198	1.12(0.99–1.27)	0.065		1.15(1.01–1.32)	0.04	
< 60	21/163	1.27(1.01–1.59)	0.038		1.37(1.03–1.81)	0.028	
History of diabetes				0.238			0.189
Yes	29/86	1.33(1.04–1.71)	0.022		1.35(1.04–1.75)	0.023	
No	59/275	1.12(0.99–1.28)	0.073		1.17(1.02–1.35)	0.03	
History of CAD				0.386			0.831
Yes	16/37	1.26(0.96–1.66)	0.096		1.15(0.79–1.68)	0.473	
No	72/324	1.15(1.02–1.29)	0.026		1.26(1.07–1.40)	0.003	
LDL-c (mmol/L)				0.398			0.246
≥ 2.45	42/181	1.12(0.95–1.33)	0.170		1.16(0.97–1.40)	0.106	
< 2.45	46/180	1.24(1.07–1.44)	0.006		1.40(1.12–1.74)	0.003	
HDL-c (mmol/L)				0.906			0.880
≥ 1.03	43/184	1.13(0.97–1.31)	0.114		1.15(0.97–1.36)	0.118	
< 1.03	45/177	1.15(0.98–1.34)	0.079		1.27(1.07–1.51)	0.008	
TG (mmol/L)				0.587			0.929
≥ 1.29	46.182	1.12(0.97–1.29)	0.117		1.21(1.03–1.42)	0.018	
< 1.29	42/179	1.19(0.99–1.44)	0.066		1.22(0.99–1.50)	0.061	

Covariates adjusted for CV death: age, dialysis vintage, vascular access, BMI, history of diabetes, coronary artery disease, albumin, hsCRP, creatine, uric acid, hs-cTNT, NT-proBNP. Covariates adjusted for MACE: age, dialysis vintage, vascular access, history of diabetes, coronary artery disease, history of taking ACEI, albumin, prealbumin, hs-CRP, creatine, uric acid, hs-cTNT, NT-proBNP

**Table 6:** Interaction analyses of Lp-PLA2 with Lipids measures in relation to CV death and MACE occurrence

CV death	*P* for interaction (unadjusted)	*P* for interaction (adjusted)
*Lp-PLA*_*2*_*(as a dichotomous variable)*		
LDL-c	0.967	0.728
HDL-c	0.638	0.631
apoB	0.635	0.965
apoA	0.803	0.336
TG	0.973	0.875
*Lp-PLA*_*2*_*(as a continuous variable)(per 100U/L)*		
LDL-c	0.721	0.4
HDL-c	0.937	0.708
apoB	0.379	0.12
apoA	0.822	0.671
TG	0.939	0.993
**MACEs**	***P* for interaction (unadjusted)**	***P* for interaction (adjusted)**
*Lp-PLA*_*2*_* (as a dichotomous variable)*		
LDL-c	0.884	0.875
HDL-c	0.634	0.948
apoB	0.681	0.928
apoA	0.615	0.665
TG	0.92	0.924
*Lp-PLA*_*2*_*(as a continuous variable)(per 100U/L)*		
LDL-c	0.909	0.98
HDL-c	0.74	0.749
apoB	0.394	0.189
apoA	0.824	0.845
TG	0.869	0.609

Covariates adjusted for CV death: age, dialysis vintage, vascular access, BMI, history of diabetes, coronary artery disease, albumin, hsCRP, creatine, uric acid, hs-cTNT, NT-proBNP. Covariates adjusted for MACE: age, dialysis vintage, vascular access, history of diabetes, coronary artery disease, history of taking ACEI, albumin, prealbumin, hs-CRP, creatine, uric acid, hs-cTNT, NT-proBNP

### Incremental prognostic value of Lp-PLA_2_ activity

The discriminative ability of models, both with and without Lp-PLA_2_ activity, was assessed using the C-index. Incorporating Lp-PLA_2_ activity into the comprehensively adjusted Cox model for CV mortality led to a marginal improvement in the C-index. Specifically, when Lp-PLA_2_ was treated as a categorical variable, the C-index rose from 0.752 (95% CI: 0.695–0.809) to 0.765 (95% CI: 0.704–0.826). Similarly, when Lp-PLA_2_ was considered as a continuous variable, the C-index was 0.766 (95% CI: 0.707–0.825). However, pairwise comparisons showed no significant differences between these models.

The results were similar in Cox model for MACE, including Lp-PLA_2_ raised the C-index from 0.745 (95% CI: 0.694–0.796), to 0.758 (95% CI: 0.705–0.811) as a categorical variable, and to 0.756 (95% CI: 0.703–0.809) as a continuous variable. Pairwise comparisons found no significant differences between the models.

NRI was assessed for Lp-PLA_2_ inclusion. As a continuous variable, the integration of Lp-PLA_2_ produced a significant NRI of 33.32% (95% CI: 7.47% to 56.21%) for CV mortality and 23.97% (95% CI: 0.28% to 46.24%) for MACE. As a categorical variable, it resulted in an NRI of 42.51% (95% CI: 5.0% to 61.33%) for CV mortality, but an inconclusive NRI of 33.0% (95% CI: -0.58% to 52.60%) for MACE.

## Discussion

This study demonstrated that among MHD patients, Lp-PLA_2_ activity exhibits a positive correlation with TC, LDL-C, triglycerides, and apolipoprotein-B, Conversely, a negative association was observed with statin usage. Crucially, increased Lp-PLA_2_ activity emerged as a predictor of CV mortality and MACE occurrence.

The task of lipid management in patients with CKD and ESKD remains clinically challenging, with patterns varying internationally [24]. The lipid profile in patients with CKD is influenced by a myriad of factors, including underlying kidney disease, kidney function, severity of proteinuria, kidney replacement therapy modality and use of medications [25–27]. While over 80% of dialysis patients exhibit dyslipidemia, their lipid disturbance patterns diverge from the general population. Notably, these patients often present with increased triglyceride and VLDL-C levels, diminished HDL-C, and, to a lesser extent, increased LDL-C and TC [28, 29]. Contrary to the evident link between serum cholesterol concentrations and LDL-C levels with the risk of CVD in subjects with a wide range of conditions, an inverse epidemiology has been observed in ESKD patients. Remarkably, prognostic outcomes appear more favorable at elevated LDL-C, HDL cholesterol, and triglyceride concentrations [30, 31]. This may arise due to misleading associations wherein reduced LDL-C levels correspond with heightened mortality risk. This can be attributed to confounders induced by kidney failure, dialysis procedures, and concomitant conditions, including malnutrition and microinflammatory states. In the dialysis population, the cardiovascular impact of increased LDL-C can be underestimated. This group exhibits a unique clinical profile wherein sudden cardiac deaths and heart failure fatalities are more prevalent than deaths directly attributed to atherosclerotic CVD [32].

In addition, in the general population, LDL-C is the key therapeutic target, as the reduction in LDL-C levels with statins is directly related to a decreased risk of major cardiovascular events [33]. Unfortunately, as glomerular filtration rates decline, the cardioprotective benefit of statins wane, with dialysis patients experiencing negligible, if any, clear advantages [13, 14, 34]. Several large-scale clinical trials in patients with CKD have confirmed these observations. In both the 4D (Die Deutsche Diabetes Dialyse) study and the AURORA (Assessment of Survival and Cardiovascular Events) trial, statin therapies, though efficacious in reducing LDL-C levels from baseline, did not demonstrate reductions in cardiovascular composite endpoints [12, 14, 35]. Further insights arise from the SHARP (Study of Heart and Renal Protection) trial, where over 9,000 CKD patients were treated with a combination of simvastatin and ezetimibe, aiming to prevent atherosclerotic vascular events [13]. The SHARP study showed promising results in non dialysis-dependent CKD patients, where lowering LDL-C with treatment reduced the risk of major atherosclerotic events, aligning with the association between LDL-C levels and CVD in this cohort. However, this benefit was absent in participants already undergoing dialysis at the enrollment. Furthermore, lipid-lowering therapy had no significant effect on all-cause mortality [13]. Upon these findings, in the KDIGO guidelines, it was recommended that statins not be initiated in dialysis patients, but they could be continued or combined with ezetimibe in those who already take them at the time of dialysis transition [34]. Given the results mentioned above, it becomes evident that, unlike in other populations, LDL-C does not serve as a consistent prognostic marker in dialysis patients, especially not in the traditionally held view that "the lower, the better" [36]. Furthermore, its role as a therapeutic target in this patient cohort remains unvalidated.

Lp-PLA_2_ is mainly secreted by macrophages. It forms complexes with LDL or HDL, especially with apolipoprotein B-containing LDL and apolipoprotein A-I-containing HDL [37]. Notably, while HDL-associated Lp-PLA2 exerts antiatherogenic effects, its LDL-bound counterpart promotes atherogenesis. [19, 37] Lp-PLA_2_ hydrolyzes oxidized LDL into two active products: lysophosphatidylcholine (lysoPC) and oxidized non-esterified fatty acids (oxNEFAs) [37–40]. The hydrolysis product lysoPC plays a major proinflammatory role by targeting endothelial cells, smooth muscle cells, monocytes/macrophages, T cells and neutrophils; affecting cellular activity, inflammatory cell homing and functional responses of endothelial and smooth muscle cells; and inducing oxidative stress and immune responses [37, 38, 40]. This process, in turn, further promotes Lp-PLA_2_ activity. Elevated Lp-PLA_2_ and lysoPC can be detected in unstable or ruptured atherosclerotic plaques but are almost nonexistent in stable plaques [39].

In this study, when stratified by median values or when assessed as a continuous variable, elevated Lp-PLA2 activity are consistently associated with an increased risk of cardiovascular mortality and the occurrence of MACE, even after adjusting for multiple confounding variables in the models. In addition, when measures of Lp-PLA_2_ and LDL-C levels were used in combination, all-cause and CV mortality were highest in the group with high Lp-PLA_2_ + low LDL-C. Notably, when analyzing Lp-PLA2 activity as a continuous variable, its significance as a prognostic marker diminished in the subset of patients with LDL-c ≥ 2.45 mmol/L, but remained robust in those with LDL-c < 2.45 mmol. These findings echoes the prior research suggesting Lp-PLA2's integral role as a prognostic risk factor in subjects exhibiting normal or reduced LDL-C levels [41]. NRI analyses also confirmed the additive prognostic value Lp-PLA_2_, especially for CV mortality, but its value can vary depending on the manner of its form (continuous or categorical) in the established predictive models. Vascular inflammation plays a critical role throughout the process of atherosclerosis [42, 43]. Patients with CKD/ESKD, burdened by the accumulation of uremic toxins, malnutrition, and associated comorbidities, endure a chronic inflammatory milieu. This state facilitates an upsurge in proinflammatory mediators, propelling the onset and progression of atherosclerosis and subsequent cardiovascular dysfunctions [2]. Lp-PLA_2_, as a bridge between inflammation and the process of atherosclerosis, may enhance the atherogenicity of LDL [38]. Given the challenges in interpreting LDL-C within the dialysis cohort, the incorporation of Lp-PLA_2_ in lipid profile assessments could offer a more nuanced approach to discerning patients with an increased cardiovascular risk.

Regarding non-linear relationship between Lp-PLA_2_ and cardiovascular outcomes, RCS analyses suggest a potential threshold at around 600–700 U/L. Up to this point, rising Lp-PLA_2_ levels seem to be associated with an increased risk of CV mortality and MACE. Beyond this threshold, the risk marginally declines, which may imply a saturation effect of Lp-PLA_2_. The confounders mentioned above may also overshadow its detrimental impact in patients with highest range of Lp-PLA_2_ activity. Further investigations could shed light on this observed trend and its clinical implications. It should be noted that the method used to assess Lp-PLA_2_ activity in this study differs from the FDA-approved method. Thus, the identified threshold cannot be directly applied based on our findings.

In the current investigation, high Lp-PLA_2_ activity was associated with high CV mortality among subjects not on statin therapy. Intriguingly, this association was attenuated among statin users. These results are consistent with the post hoc analysis by Winkler et al. for the 4D study, wherein a discernible association between Lp-PLA_2_ activity and CV events emerged solely in statin-naïve patients [22]. It is essential to underscore the limitations presented by the sample size, which restricts the capacity to definitively establish the utility of Lp-PLA_2_ in identifying those who might benefit from statin use. There is a pressing need for an in-depth exploration of Lp-PLA_2_ in the dialysis cohort to elucidate its clinical utility, particularly in identifying patients poised to derive maximal benefits from statin therapy.

Two large, randomized trials involving patients with CAD investigated Lp-PLA_2_ as a potential therapeutic target, administering an Lp-PLA_2_ inhibitor in addition to the standard treatment. [44, 45] In both trials, adding Lp-PLA_2_ inhibitors to optimal medical treatment failed to reduce the risk of major coronary events. The high level of standard of care implemented in these trials may minimize the residual risk in clinical trials and overshadow any incremental benefits conferred by Lp-PLA_2_ inhibition. Intriguingly, subgroup analyses did suggest that cigarette smokers—individuals subjected to increased inflammation and oxidative stress—might experience therapeutic advantages from Lp-PLA_2_ inhibition. Given that ESKD patients are also in a state of chronic inflammation, similar to smokers, it is conceivable that they might similarly benefit from Lp-PLA_2_ inhibition. The persistent inflammation in ESKD patients and its profound interaction in atherosclerosis may be more important than the absolute level of LDL-C. With the vast unmet need regarding lipid management in ESKD patients, it remains prudent not to dismiss the prospective utility of Lp-PLA_2_ as an emergent therapeutic paradigm. Nevertheless, considering the current findings, future research should mainly pivot towards exploring its role as a stratification tool in ESKD patients. This includes broadening the scope of investigation to encompass other renal replacement modalities (e.g. peritoneal dialysis, kidney transplantation). In addition, future studies should explore the comparative clinical utility of Lp-PLA_2_ between dialysis patients and those with non-dialysis CKD, and examine the trajectory of Lp-PLA_2_ in longitudinal studies, so as to understand the long-term implications of elevated Lp-PLA2 activity, particularly across different stages of CKD.

### Comparisons with other studies and what does the current work add to the existing knowledge

To the best of our knowledge, this 7-year cohort study is the first to demonstrate the association between Lp-PLA_2_ activity and CV death in MHD patients. Prior research has underscored the proinflammatory and proatherosclerotic roles of Lp-PLA_2_, revealing its elevated levels to be concomitant with an increased risk of CAD, stroke, and mortality [18–20]. For hemodialysis recipients, existing literature has associated Lp-PLA_2_ with carotid stenosis and peripheral arterial disease [46]. A post hoc analysis of the 4D study identified high Lp-PLA_2_ activity as a predictor of CV events and mortality in diabetic MHD patients [22]. Mauri et al. found higher levels of Lp-PLA_2_ to be an independent risk factor for acute CV events in MHD patients but failed to demonstrate an association with CV death [21].

ESKD patients, owing to their intricate internal milieu coupled with associated ailments like malnutrition, are different from the general populace, thereby diminishing the efficacy of conventional risk factors. Therefore, the present research combined Lp-PLA_2_ and LDL-C to predict CV mortality, MACE occurrence and all-cause mortality in MHD patients. Patients with high Lp-PLA_2_ + low LDL-C had the highest CV mortality, further supporting the reverse epidemic phenomenon of the lipid profile and underlining the pivotal role of inflammation in CVD onset and progression in MHD patients. This study provides new insight into the clinical evaluation of CV risk and lipid management in the MHD population.

### Study strengths and limitations

The primary strength of this study lies in its extended follow-up duration (7 years), which affirmed the prognostic value of Lp-PLA_2_ for CV death and MACE in MHD patients.

Several limitations must be noted. First, initiation or discontinuation of lipid-lowering therapy was not recorded, and serial measurements of Lp-PLA_2_ and other lipid parameters were not conducted. The baseline measurements may not accurately present the exposure. Second, detailed nutritional parameters and quantified scoring assessment were not available due to constraints of the dataset. Impact of malnutrition on Lp-PLA_2_ and other lipids profile was not evaluated. comprehensive nutritional assessments would provide valuable insights into the intertwined roles of malnutrition, Lp-PLA_2_ activity, and cardiovascular risks in hemodialysis patients. Third, due to regional differences in hemodialysis practice, it is imperative to validate the results from this single-center study in an external population.

## Conclusions

In summary, this study revealed a significant association between increased Lp-PLA_2_ activity and an increased risk of CV mortality and MACE occurrence in MHD patients, even after comprehensive adjustment for both conventional and unconventional risk factors. These results bolster the potential utility of Lp-PLA_2_ as a valuable prognostic factor for cardiovascular outcomes. Furthermore, the integrated analysis of Lp-PLA_2_ and LDL-C augments prognostic precision, potentially aiding clinicians in identifying patients at a higher risk of CV events, thereby influencing clinical strategies and interventions.

### Supplementary Information

**Additional file 1: Fig. S1.** Kaplan‒Meier curves of (A) all-cause mortality, (B) CV mortality and (C) MACEs stratified by medians of both Lp-PLA_2_ and LDL-C in non-statin users.

**Additional file 2: Fig. S2.** Kaplan‒Meier curves of (A) all-cause mortality, (B) CV mortality and (C) MACEs stratified by medians of both Lp-PLA_2_ and LDL-C in statin users.

## Data Availability

The datasets used and/or analyzed during the current study are available from the corresponding author on reasonable request.

## Abbreviations

CVD: Cardiovascular disease
LDL-C: Low-density lipoprotein cholesterol
Lp-PLA_2_: Lipoprotein-associated phospholipase A2
apo: Apolipoprotein
NT-proBNP: N-terminal pro b-type natriuretic peptide
hs-cTnT: High-sensitivity cardiac troponin T
hs-CRP: High-sensitivity C-reactive protein
CKD: Chronic kidney disease
CAD: Coronary artery disease
ESKD: End-stage kidney disease
CV: Cardiovascular
MHD: Maintenance hemodialysis
VLDL-C: Very low-density lipoprotein cholesterol
HDL-C: High-density lipoprotein cholesterol
LDL: Low-density lipoprotein
Hcy: Homocysteine
CVA: Cerebral vascular accident
4D: Die Deutsche Diabetes Dialyse
AURORA: Assessment of Survival and Cardiovascular Events
SHARP: Study of Heart and Renal Protection
HDL: Lysophosphatidylcholine
oxNEFAs: Oxidized non-esterified fatty acids
