# Supplementary figures and images for: Lipoprotein-associated phospholipase A2 predicts cardiovascular death in patients on maintenance hemodialysis: a 7-year prospective cohort study
Source: Lipids Health Dis. 2024 Jan 12;23:15. doi: 10.1186/s12944-023-01991-0 (PMC10785463; doi:10.1186/s12944-023-01991-0)

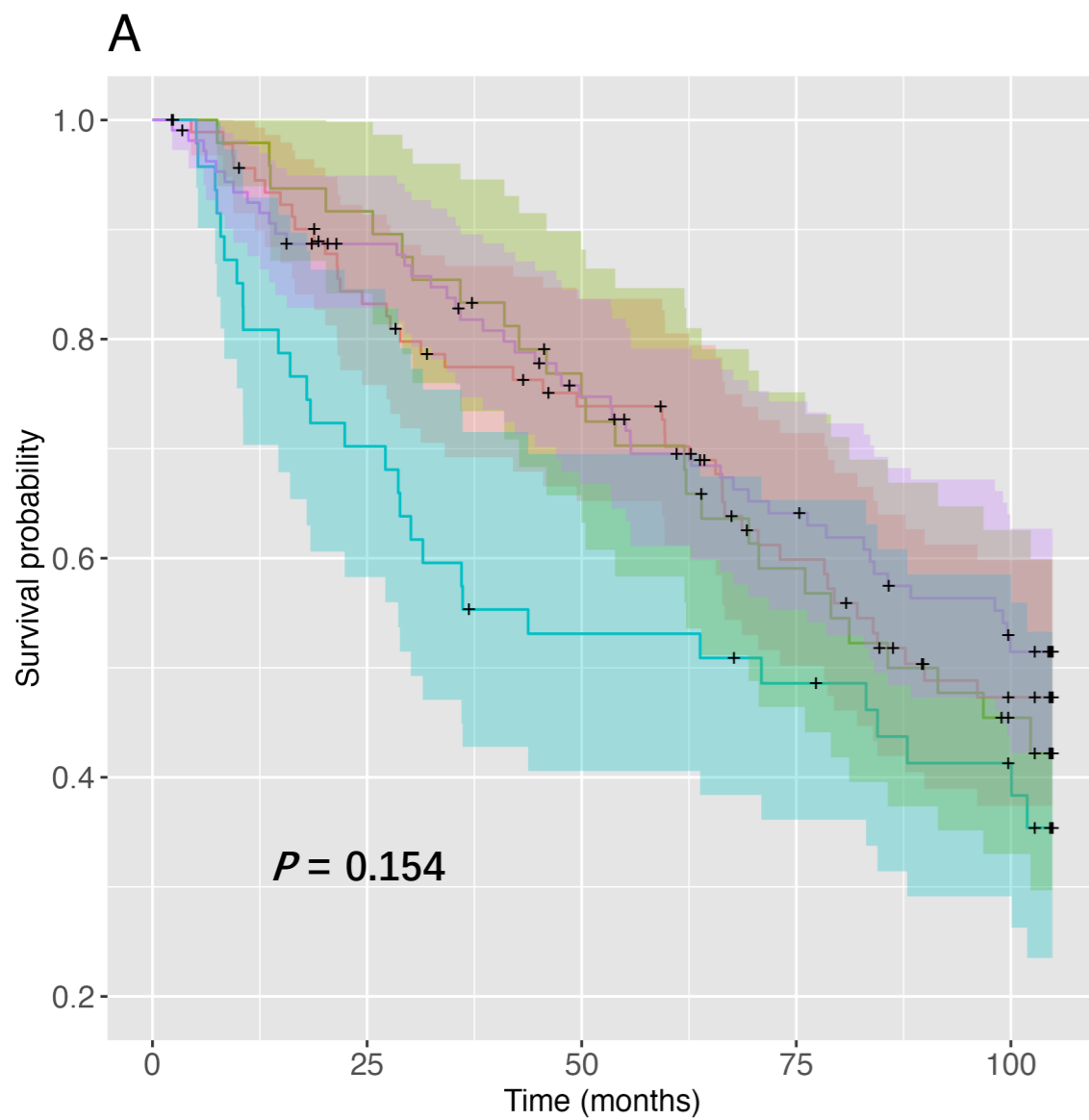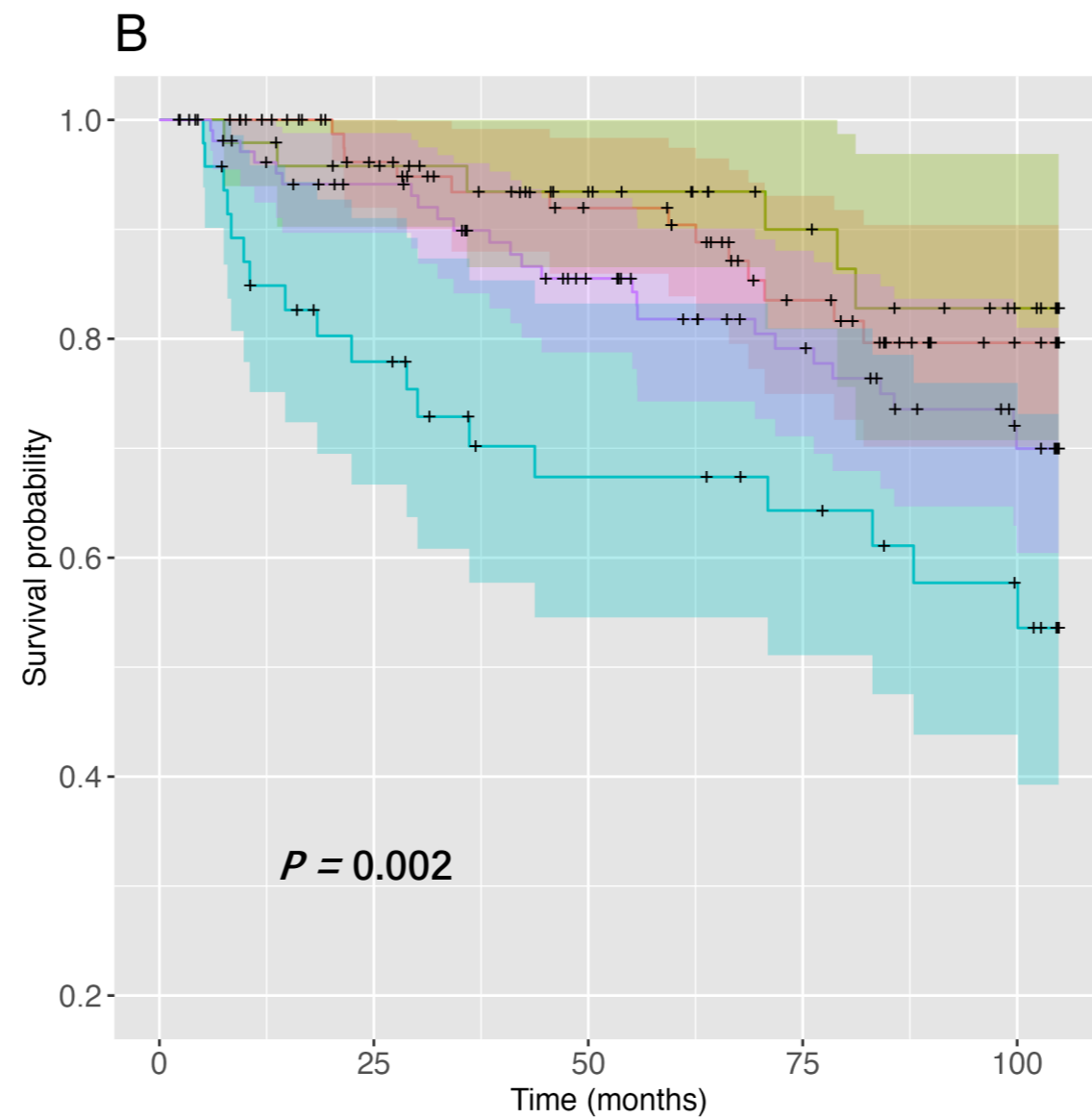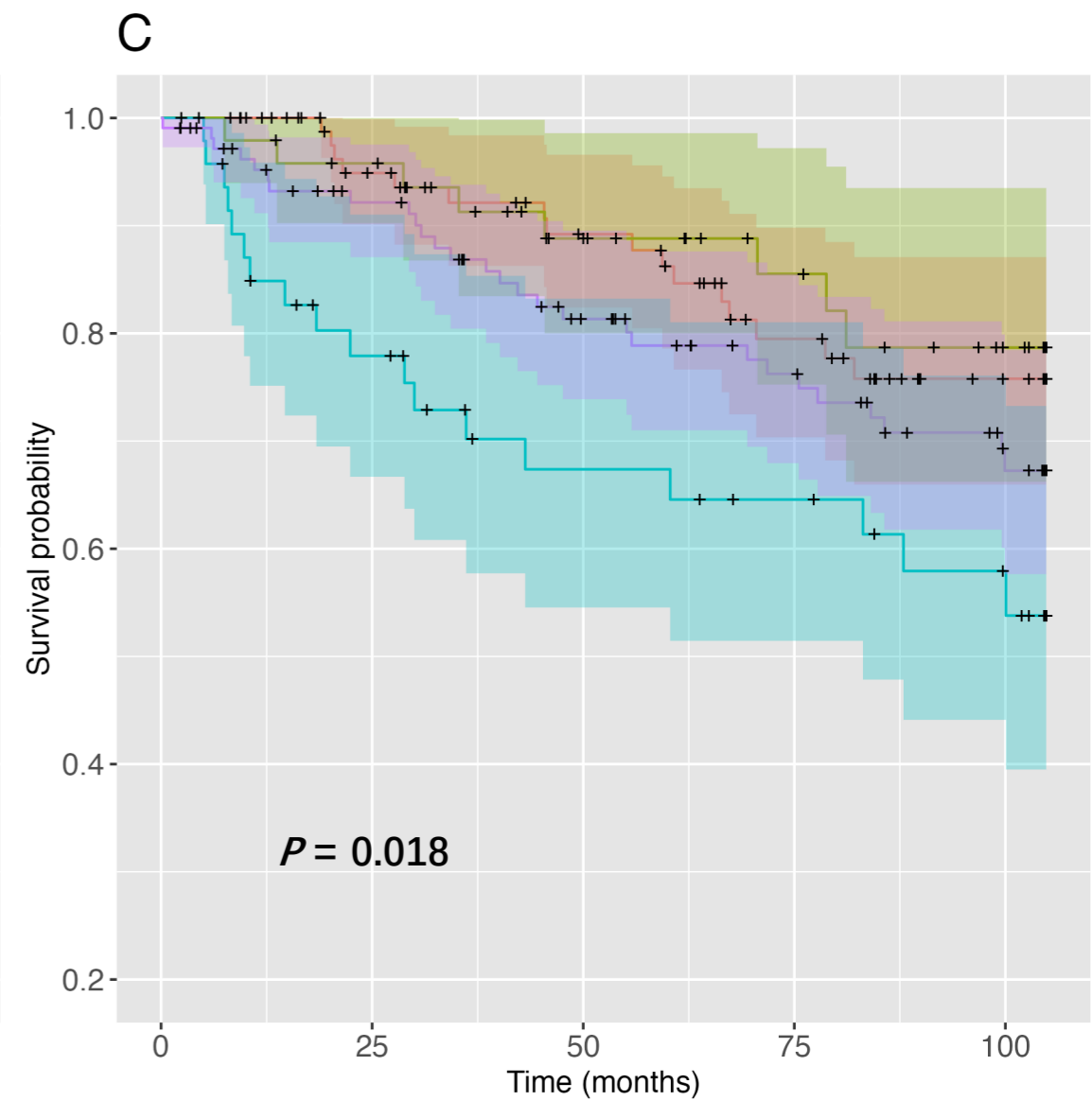

Supplement: Supplementary file 1 — Additional file 1: Fig. S1. Kaplan‒Meier curves of (A) all-cause mortality, (B) CV mortality and (C) MACEs stratified by medians of both Lp-PLA2 and LDL-C in non-statin users. [file 12944_2023_1991_MOESM1_ESM.pdf]

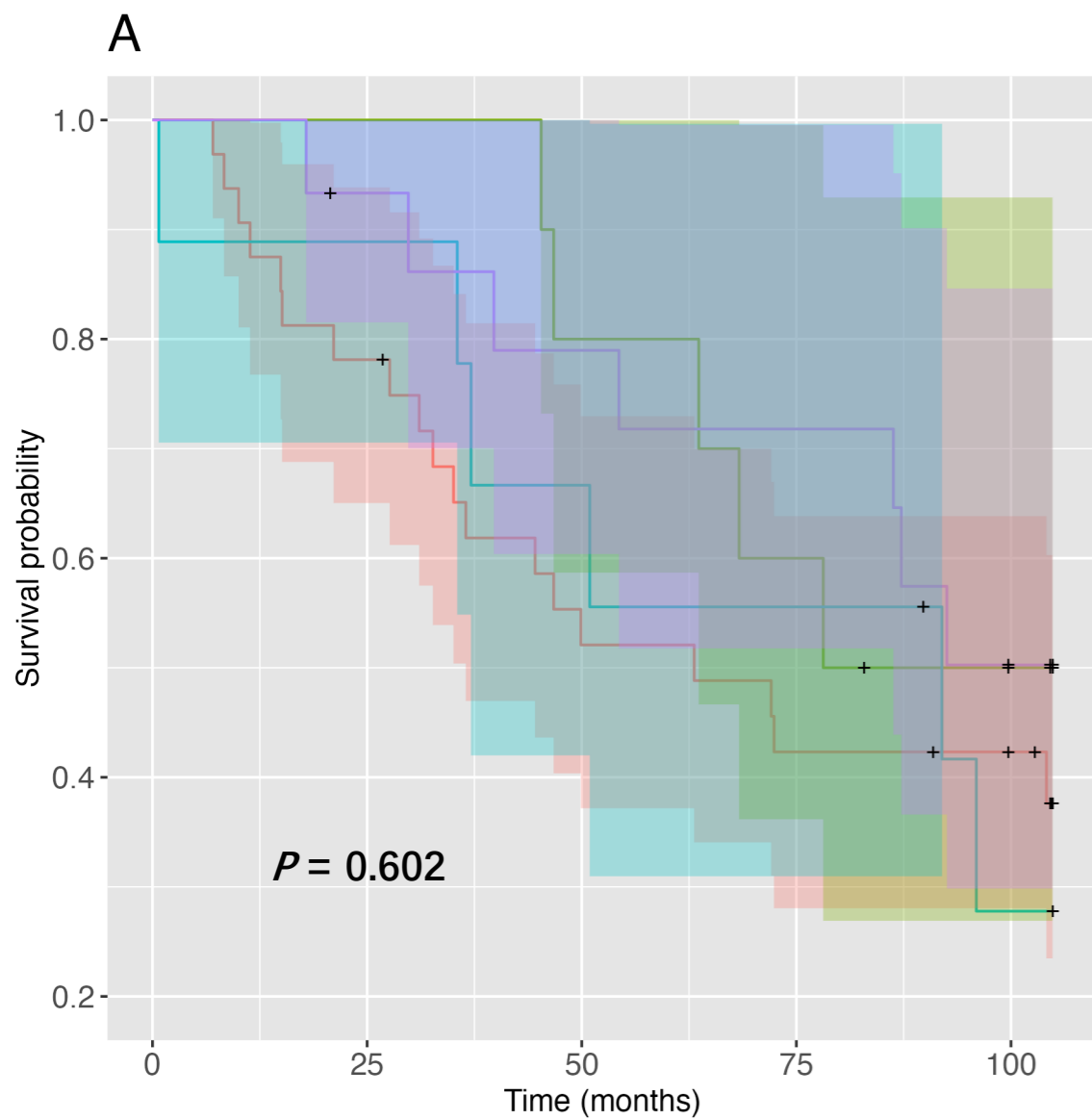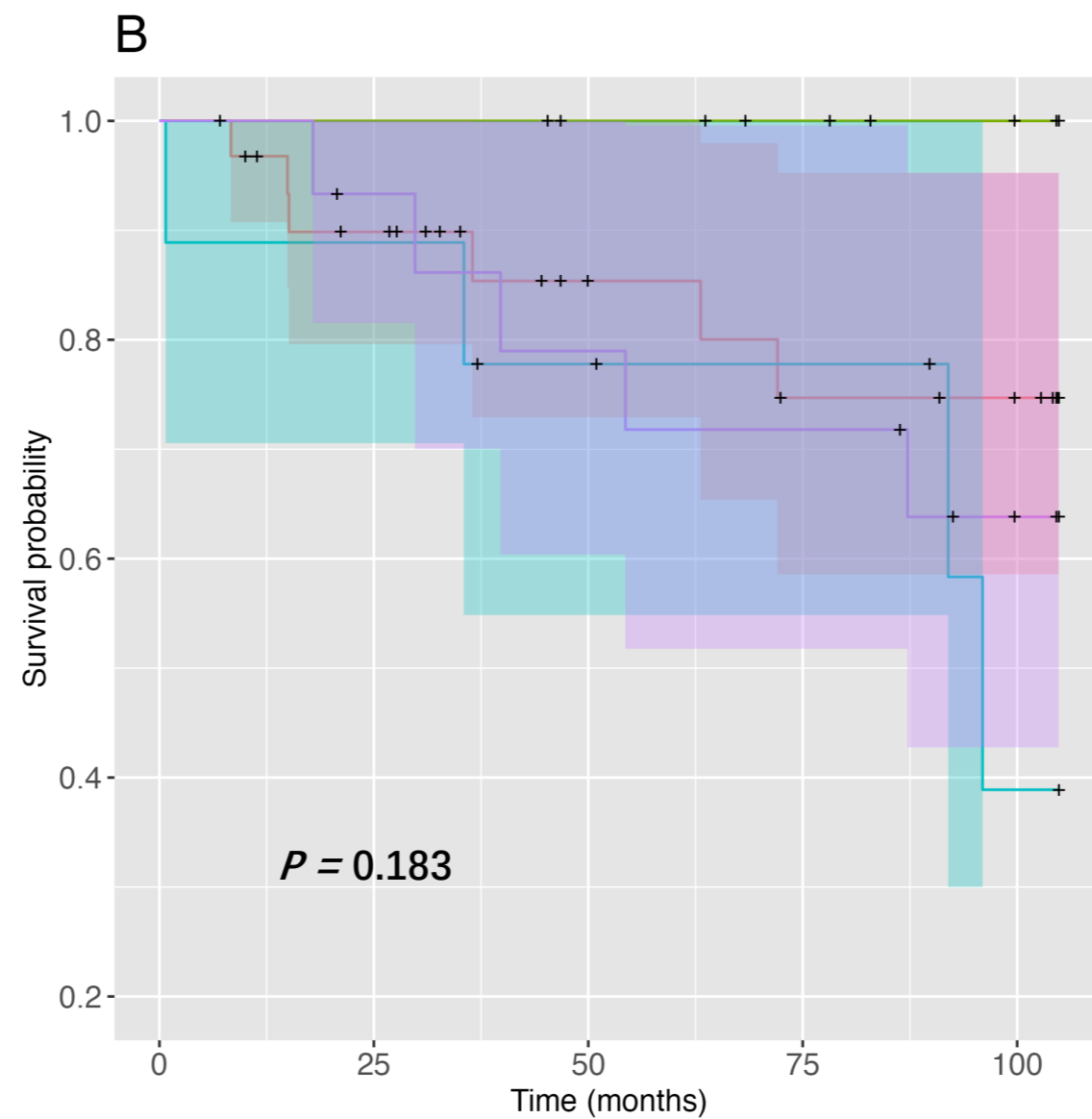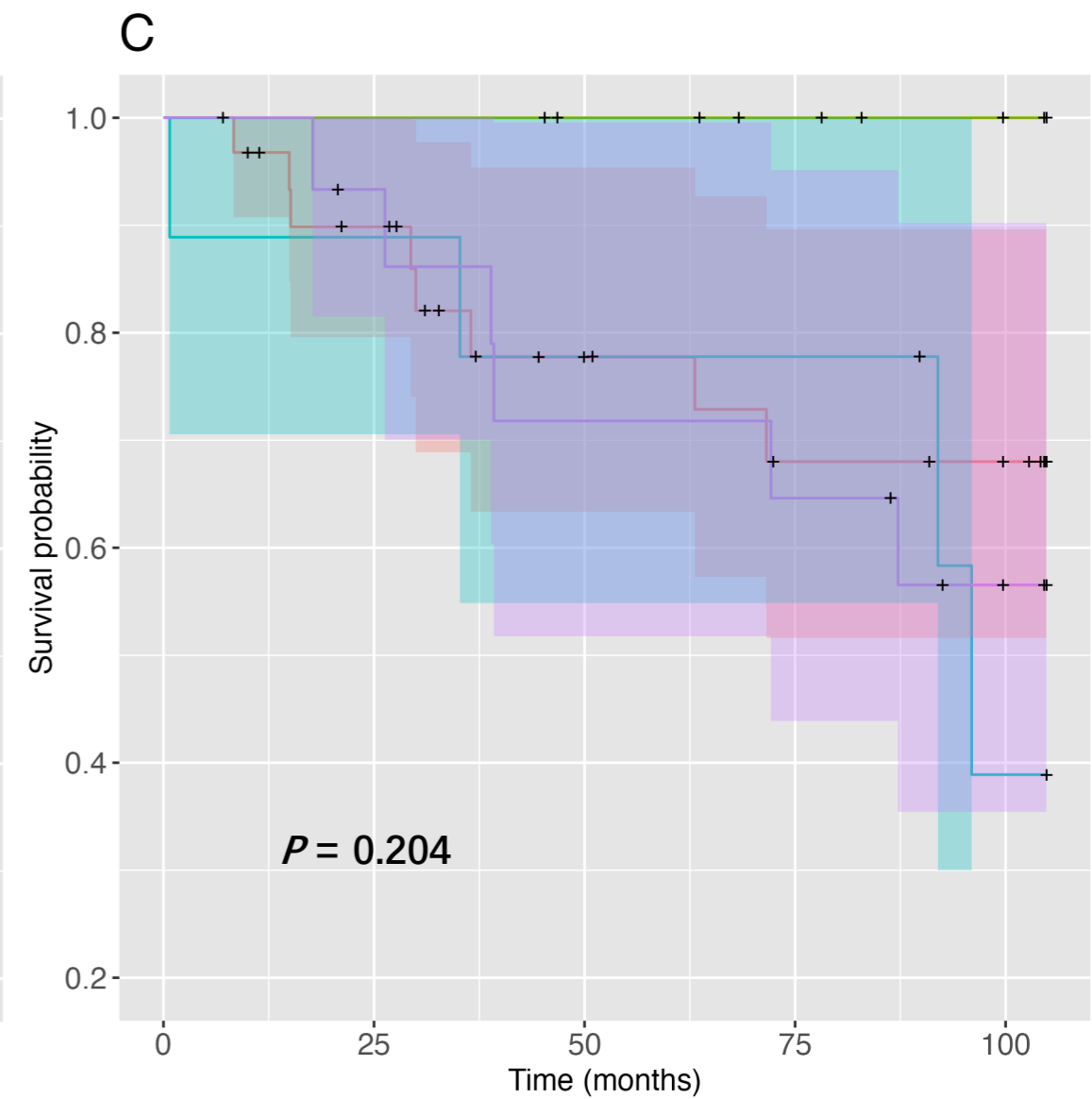

Supplement: Supplementary file 2 — Additional file 2: Fig. S2. Kaplan‒Meier curves of (A) all-cause mortality, (B) CV mortality and (C) MACEs stratified by medians of both Lp-PLA2 and LDL-C in statin users. [file 12944_2023_1991_MOESM2_ESM.pdf]
